# EFFECTIVENESS OF VIRTUAL REALITY ASSISTED ACTIVE LIMB MOVEMENT EXERCISES FOR PATIENTS IN THE RESPIRATORY INTENSIVE CARE UNIT: A RANDOMIZED PILOT STUDY

**DOI:** 10.2340/jrm.v57.28399

**Published:** 2025-06-03

**Authors:** Jiani WANG, Chenxi SHI, Yanrui JIA, Qian XIAO

**Affiliations:** 1 Fuwai Hospital, CAMS & PUMC, Beijing, China; 2 Department of Respiratory and Critical Care Medicine, Beijing Institute of Respiratory Medicine and Beijing Chao-yang Hospital, Capital Medical University, Beijing, China; 3 Beijing Chaoyang Hospital affiliated to Capital Medical University, Beijing, China; 4 School of Nursing, Capital Medical University, Beijing, China

**Keywords:** early limb mobilization, exercise compliance, virtual reality rehabilitation, respiratory intensive care, muscle strength improvement, RICU patient outcomes

## Abstract

**Objective:**

The primary aim of this study is to compare the effectiveness of early active limb movement facilitated by virtual reality technologies with conventional exercise therapy in enhancing patient recovery in the Respiratory Intensive Care Unit. The follow-up period covers 1 week.

**Methods:**

In this prospective randomized controlled trial, patients were allocated to either a control group, which received standard exercise therapy, or a virtual reality group, which utilized virtual reality software and equipment for active exercises. Patients were followed for 1 week. The study compared compliance, safety, and rehabilitative outcomes between these groups. Data were analysed using a linear mixed-effects model.

**Results:**

Patients in the virtual reality-based exercise group exhibited significantly higher levels of average daily exercise time, out-of-bed exercise time, and overall exercise compliance compared with the control group (*p* < 0.05). There were no reports of adverse events related to exercise in either group. Notably, within the first week of intervention, the virtual reality-based exercise group showed significant improvements in various parameters, including muscle strength, grip strength, body mass index, and the Barthel Index, outperforming the control group in these areas (all *p* < 0.05).

**Conclusion:**

Based on 1 week of follow-up data, the study confirms that virtual reality-based exercise modalities are more efficacious than traditional exercise approaches in enhancing exercise duration, compliance, and various health outcomes in Respiratory Intensive Care Unit patients. This approach also contributes to reducing Respiratory Intensive Care Unit stay duration. The system’s effectiveness could be further increased by integrating more varied and engaging rehabilitation games and features tailored to the needs of ICU patients.

*Trial registration*: Chictr.org: ChiCTR1900021452

LAY ABSTRACT

This study explored whether using virtual reality technology to help patients in the Respiratory Intensive Care Unit perform early limb exercises could improve their recovery compared to traditional exercise methods. Patients were divided into two groups: one used virtual reality to assist with exercises, while the other followed standard exercise routines. Over one week, the virtual reality group showed better results in terms of exercise time, muscle strength, grip strength, and overall recovery. The study found that virtual reality-based exercises were more engaging and effective, leading to higher patient compliance and faster improvements in physical health. This suggests that virtual reality could be a valuable tool in helping Respiratory Intensive Care Unit patients recover more quickly and efficiently.

Intensive Care Unit (ICU) early mobilization is widely recognized as a safe and effective rehabilitation therapy that can enhance ICU patients’ exercise ability, awareness, and quality of life (1, 2). Despite the call for more significant efforts to implement early mobilization in clinical settings, the situation remains concerning. For instance, a multicentre survey conducted in Australia and New Zealand revealed that only 37% of patients could move out of bed, and none of the mechanically ventilated patients (43.2%) could do so (3). Similarly, a survey in Germany found that out-of-bed activities were performed by only 24% of the 783 patients (4). In the United States, less than one-third of patients with acute respiratory failure received early mobilization treatment (5).

The development rate of early mobilization in China reported that, in comprehensive ICUs, the proportion of mechanically ventilated and non-mechanically ventilated patients who underwent early activity was approximately 19.15% and 23.5%, respectively (6). Another study reported that among 548 ICU cancer patients eligible for early mobilization, only 40% received it (7). In Sweden, a survey conducted in 35 ICUs revealed that passive mobilization was the predominant approach, with only 33% of patients experiencing active mobilization (8). Similarly, in a survey of 11 ICUs in southern Brazil, only 10% of mechanically ventilated patients and 2% of patients with endotracheal intubation engaged in out-of-bed activity (9). These findings highlight the discrepancy between the potential benefits of early mobilization and its limited implementation in clinical practice. The implementation rate of early mobilization in the ICU, particularly active mobilization, has been low. Patients face difficulties persisting with mobilization due to factors such as the nature of their disease and psychological barriers (10, 11). In recent years, researchers have proposed using information technology to improve patient compliance and increase the quantity of mobilization (12, 13). One such technology is virtual reality (VR), which has not been widely used in the rehabilitation of critically ill patients. Kho et al. reported the feasibility of using an interactive video game to replace traditional early mobilization (14). This was followed by a study conducted by Abdulsatar et al. in 2013, where 12 children on mechanical ventilators participated in Nintendo WiiTM boxing exercises, significantly increasing upper limb activity without adverse events (15). Studies by Sara Parke et al. demonstrated high acceptance of VR mobilization among patients (16). However, the effectiveness of the VR exercise mode for rehabilitation in ICU patients, especially those in the Respiratory Intensive Care Unit (RICU) who require long-term ventilation support, remains unknown (17). Therefore, this study aims to assess the safety and effectiveness of VR exercise mode in RICU patients.

## METHODS

### Trial design

This study utilized a pilot single-centre clinical randomized controlled trial design. The trial occurred in a Respiratory Intensive Care Unit (RICU) with 16 beds in Beijing. The study period spanned from March 2019 to January 2020. Patients were followed up for the duration of their ICU stay and hospital stay, and outcomes were measured at the end of hospitalization.

### Participants

The inclusion criteria for participants were as follows: (1) patients in the RICU with severe respiratory diseases and aged 18 years or older; (2) patients not undergoing extracorporeal membrane oxygenation (ECMO) treatment; (3) patients with a consciousness state RASS > –1; (4) patients receiving respiratory support with FiO_2_ < 0.6 and peep < 10 cmH_2_O; (5) patients who had not increased the dose of vasoactive drugs for more than 2 h; (6) patients with arrhythmias without active myocardial ischaemia and not receiving drug treatment; (7) patients without activity contraindications, such as unstable fractures.

Exclusion criteria were as follows: (1) patients with limb loss or paralysis, binocular blindness, or other conditions that prevented active limb movement; (2) patients whose condition worsened, who died, or were transferred within 3 days, thus not meeting the requirement for active limb movement; (3) patients who had participated in other studies that could have influenced the implementation of this study and the accuracy of its results.

### Randomization and blinding

A staff member who was not involved in the study conducted the randomization process in Microsoft Excel (Microsoft Corp, Redmond, WA, USA), ensuring that subjects were randomly allocated to the VR-EM and the control groups. Randomization was conducted using a 1:1 allocation ratio to achieve balanced group sizes. The final group sizes were the VR group (*n* = 33) and control group (*n* = 35), with minor variations in numbers due to practical constraints such as patient exclusion or withdrawal during enrolment. The random grouping sequence was recorded in sealed opaque envelopes, ensuring that the distribution of the groups remained concealed. Following the collection of baseline data, the envelopes were opened in the order of enrolment to reveal the grouping information of the patients.

### Interventions

Patients in the intervention group utilized a self-developed early mobilization rehabilitation system for RICU patients, the VR-EM system (18). This system assisted patients in performing active limb movements through human–computer interaction. The mobilization activities included a range of movements such as plane movements, fist clenching, wrist extensions, elbow flexions, shoulder abductions, resistance exercises using dumbbells and resistance bands, hip flexions, knee extensions, ankle dorsal flexions and extensions, active straight leg abductions, sitting training, and assisted standing and walking (Table I). The VR-EM system targeted both gross movements of large joints and delicate movements of minor joints. The VR group engaged in active limb movements facilitated by the VR system, which included interactive and immersive exercises such as virtual piano playing for wrist stretches and virtual games for shoulder and elbow movements.

**Table I:** Description of the intervention and virtual reality (VR) technology

	Control group	VR-EM group
Intervention	Standard rehabilitation exercises: passive limb movements, assisted standing, and walking	Active limb movements facilitated by the VR system: interactive and immersive exercises
Motivating Participants	Establish a trusting relationship with patients, maintain communication with participants, and give timely feedback	
Participant motivation	Improve outcome	
VR technology	VR-EM system assisted patients in performing active limb movements through human–computer interaction The mobilization activities included a range of movements such as plane movements, fist clenching, wrist extensions, elbow flexions, shoulder abductions, resistance exercises using dumbbells and resistance bands, hip flexions, knee extensions, ankle dorsal flexions and extensions, active straight leg abductions, sitting training, and assisted standing and walking	

Establishing a trusting relationship, maintaining effective communication, and providing timely feedback during rehabilitation enhanced participant motivation in the VR group. These strategies aimed to foster engagement and adherence to the prescribed exercise regimen (detailed in Table I).

On the other hand, patients in the control group followed a traditional exercise regimen. The control group performed standard rehabilitation exercises under the supervision of a nurse or respiratory therapist, including activities such as passive limb movements, assisted standing, and walking. Both groups were required to perform exercises for 20 min, 3 times a day in the morning, afternoon, and evening. Adherence to exercise was assessed by monitoring the consistency and completeness of the prescribed exercise sessions, with “good” adherence indicating that patients consistently performed the exercises as instructed and were highly engaged in their rehabilitation.

### Outcome measures

The leading indicators collected include the compliance, safety, and rehabilitation indexes. Compliance indicators include exercise time, type, and patient compliance performance. Exercise time included in-bed exercise and out-of-bed exercise. Patient compliance performance was judged by adherence to the exercise. Safety indicators focus on exercise-related adverse events and the reasons for failure to exercise normally. Adverse events mainly refer to the adverse reactions caused by exercise, including tracheal loosening and falling. The reasons for failure to exercise normally included subjective and objective reasons. Subjective reasons mainly refer to the patient’s willingness to refuse to exercise. Objective reasons include disease changes, detection, operation, etc., which cause patients to be unable to exercise. Rehabilitation indicators encompass muscle strength, grip strength, handgrip strength, body mass index, and Barthel Index. We used the MRC score for muscle strength measurements and a handgrip dynamometer for grip strength measurements. All the outcomes of the indicators were recorded by medical staff, who had the required qualifications and training to carry out the study.

### Statistical analysis

All data were analysed using SPSS software (version 22.0, IBM Corp, Armonk, NY, USA). Normality tests were conducted on all continuous data. Data following a normal distribution were presented as mean and standard deviation, while non-normally distributed data were expressed as median and interquartile range. Counting data were statistically described using the percentage composition. The independent sample t-test or rank sum test was used to compare the measurement data between the virtual reality and control groups. The χ^2^ or accuracy test was employed to compare the rates or percentage compositions of the 2 groups. A linear mixed model was utilized for analysing repeated measurements.

## RESULTS

### Patient enrolment, allocation, and baseline data comparison in RICU VR study

Eighty-two patients who met the inclusion criteria were initially considered during the study. However, 14 patients were subsequently excluded based on the exclusion criteria. The reasons for exclusion were as follows: 6 patients had an expected duration of RICU hospitalization of less than 3 days, 4 patients were unable to perform active limb movements, 3 patients were concurrently involved in other clinical studies that could potentially impact the results of this study, and 1 patient declined to participate. Ultimately, 68 patients were enrolled; 33 were assigned to the virtual reality group and 35 to the control group.

**Fig. 1:**
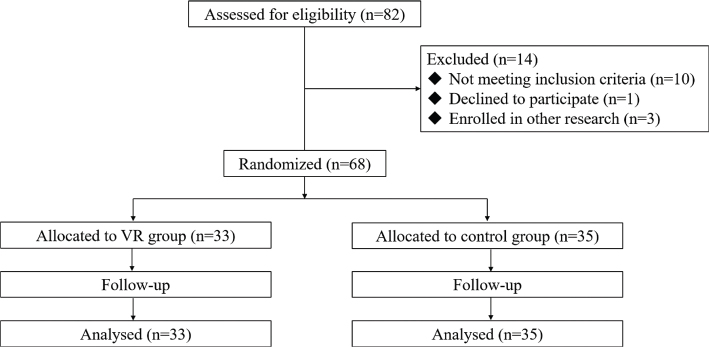
Patient enrolment flowchart for the RICU VR study.

Each patient was administered early mobilization in the RICU for at least 3 days, and no subjects were lost to follow-up during the study (Fig. 1). Baseline data, including general information and disease status, were collected and compared between the 2 groups. The results indicated no statistically significant differences in these characteristics, ensuring comparability between the virtual reality and control groups. Detailed comparison results are provided in Table II.

**Table II:** Comparison of general data, disease condition, and baseline data between the 2 groups

Factor	VR-EM group (*n* = 33)	Control group (*n* = 35)	*t*/*Z*/*χ*^2^	*p*-value

	(x ± s)/M(Q1~Q3)/n(%)			
Gender				
Male	18 (54.5)	23 (65.7)	0.885^c^	0.347
Female	15 (45.5)	12 (34.3)		
Age (years)	61 (40~70)	56 (45~73)	519^b^	0.877
BMI (kg/m^2^)	24.0±4.5	24.6±4.0	2.61^a^	0.623
Education level				
Junior high school and below	10 (30.3)	8 (22.9)	1.940^c^	0.820
High school/technical secondary school	16 (48.5)	15 (42.9)		
Junior college	3 (9.1)	5 (14.3)		
Bachelor degree and above	4 (12.1)	7 (20.0)		
Job type				
Brainpower	3 (9.1)	6 (17.1)	1.284^c^	0.609
Labour force	7 (21.2)	5 (14.3)		
Mix brain and labour	23 (69.7)	24 (68.6)		
APACHE II score	7 (5~9)	8 (5~10)	570.5^b^	0.392
Charlson co-disease index	2 (1~2.5)	2 (1~4)	618.5^b^	0.123
Respiratory support type				
Invasive	19 (57.6)	22 (62.8)	0.120^c^	0.792
Non-invasive	14 (42.4)	13 (37.2)		
Braden pressure sore risk score				
High risk	10 (30.3)	12 (34.3)	1.280^c^	0.527
Medium risk	13 (39.4)	12 (34.3)		
Low risk	10 (30.3)	11 (31.4)		
Johns Hopkins risk score for falling off the bed				
High risk	6 (18.2)	7 (20.0)	0.190^c^	0.945
Medium risk	20 (60.6)	22 (62.9)		
Low risk	7 (21.2)	6 (17.1)		
Unplanned extubation risk score				
High risk	10 (30.3)	13 (37.1)	3.635^c^	0.057
Low risk	23 (69.7)	22 (62.9)		
MRC score baseline	46 (43~52)	48 (48~54)	572.5^b^	0.373
ICUAW rate				
Yes	11 (33.3)	11 (31.4)	0.297^c^	0.586
No	22 (66.7)	24 (68.6)		
Grip strength (kg)	12 (7~22)	13 (9~22)	541.5^b^	0.647
Grip strength/weight index (%)	15 (11~32)	15 (11~33)	509.5^b^	0.978
Barthel Index	20 (5~40)	20 (5~60)	560.0^b^	0.474

a *t*-value,

b *Z* value,

c *χ*^2^ value.

BMI: body mass index, BMI = weight(kg)/height (metre)^^2^; APACHE II score: Acute Physiology and Chronic Health Evaluation II score, a severity of disease classification system used to predict the risk of death or other adverse outcomes for patients in intensive care units; MRC score: Medical Research Council score, a standardized method for assessing muscle strength; ICUAW: Intensive Care Unit-Acquired Weakness, a condition characterized by the development of muscle weakness in patients during their stay in the intensive care unit.

### Comparative analysis of exercise compliance indicators in VR-EM and control groups

Compliance indicators were compared between the 2 groups, including the average exercise days, average total daily exercise time, average daily exercise time in bed, average daily exercise time out of bed, proportion of exercise in and out of bed, and exercise compliance. Table III shows that the total daily exercise time, average daily out-of-bed exercise time, and exercise compliance in the VR-EM group significantly differed from those in the control group (all *p* < 0.05). The VR-EM group performed more engaging exercises and provided real-time feedback, which differed from the standard exercises given to the control group.

**Table III:** Comparison of exercise days, exercise times, and adherence between the 2 groups

Factor	VR-EM group (*n* = 33)	Control group (*n* = 35)	*t*/*Z*/*χ*^2^	*p*-value

	(x ± s)/M(Q1~Q3)/n(%)			
Exercise days	5 (3.5~9)	6 (4~8)	537.5^b^	0.368
Average total daily exercise time (min)	56.6±17.9	22.4±19.0	6.839^a^	**0.001**
Average length of daily in-bed exercise time (min)	15 (3~32)	8 (0~22)	170.5^b^	0.208
Average length of daily out-bed exercise time (min)	19 (0~36)	5 (0~48)	111.0^b^	**0.005**
Exercise form				
In-bed exercise	16 (48.5)	20 (57.1)	1.455^c^	0.228
Out-of-bed exercise	17 (51.5)	15 (42.9)		
Adherence to exercise				
Good	7 (21.2)	1 (2.8)	13.17^c^	**0.001** ^d^
Middle	21 (63.6)	19 (54.3)		
Weak	5 (15.2)	15 (42.9)		

a *t*-value,

b *Z* value,

c *χ*^2^ value,

d represents 2 groups of patients’ exercise compliance evaluation by *χ*^2^ division pairwise comparison, the 2 groups of patients’ exercise compliance evaluation as good and medium constituent ratio difference is statistically significant (adjusted *p* = 0.009), the 2 groups of patients’ exercise compliance evaluation as good and poor constituent ratio difference is statistically significant (adjusted *p* = 0.005). The items shown in bold indicate statistically significant differences between the VR-EM group and the control group (p < 0.05). These results highlight the areas where the VR-based exercise intervention had a measurable and significant impact compared to the traditional exercise methods.

### Exercise interruptions and safety observations in RICU patients undergoing VR and control interventions

During the study period, intermittent exercise suspensions were primarily caused by subjective reasons, such as patients or their family members feeling tired or uncomfortable. Vital signs were closely monitored during all exercise sessions to ensure patient safety. Only 3 instances of external trachea loosening were recorded, and the medical staff managed them promptly. No other exercise-related adverse reactions or safety incidents were reported. There were 41 instances of exercise suspension, including 18 in the virtual reality group and 23 in the control group. Additionally, there were 21 instances where exercise was not carried out for objective reasons, such as changes in illness or the need for examination. Of these, 12 instances occurred in the virtual reality group and 9 in the control group. The causes of exercise pause did not significantly differ between the 2 groups (*p >* 0.05).

Throughout the study period, the patient’s vital signs, including heart rate, respiration, blood oxygen saturation, and blood pressure, fluctuated within ± 10% before and during exercise. Bedside nurses or respiratory therapists were present during the intervention to ensure patient safety. Only 3 times during exercise did the external trachea loosen and fall off, but the researchers and bedside healthcare professionals promptly reconnected the tube. No exercise-related adverse reactions or safety incidents were observed (all *p* > 0.05).

### Comparative trends in muscle and grip strength and BMI between VR-EM and control groups over one week

**Fig. 2:**
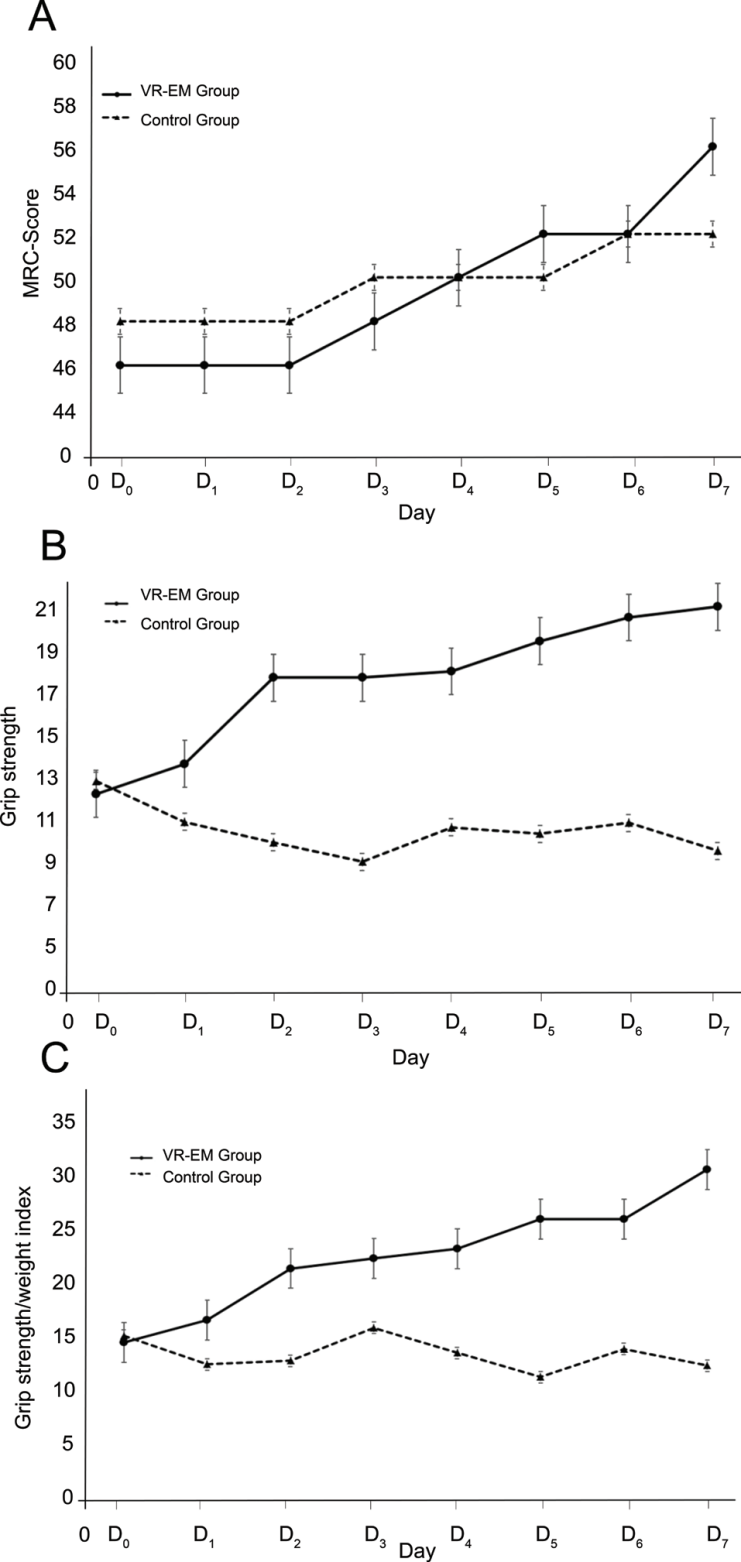
Trend analysis of muscle, grip strength, and BMI in VR-EM vs control groups. (A) Daily changes in muscle strength scores (MRC scores) in the VR-EM and control groups after intervention. (B) Daily changes in grip strength in the VR-EM and control groups after intervention. (C) Daily changes in grip strength-to-weight ratio in the VR-EM and control groups after intervention.

A comparison was made between the 2 groups regarding the changing trends in muscle strength (MRC score), grip strength, and grip strength/weight index at baseline (D0) and within 1 week of intervention (D0~D7). A linear mixed model was used to analyse these trends statistically. Fig. 2A–C illustrates that the VR-EM group had higher values at baseline and after 1 week of intervention compared with the control group, suggesting that the VR-EM group might have experienced a faster recovery in these rehabilitation indicators than the control group.

The statistical analysis showed that muscle strength (MRC score) was significantly higher in the VR-EM group compared with the control group (F = 7.921, *p* = 0.006), and the interaction analysis revealed that the effect of VR intervention persisted and strengthened over time (F = 50.10, *p* < 0.001). For grip strength, there was no significant difference between the 2 groups (F = 1.430, *p* = 0.235); however, significant improvements were observed over time within both groups (F = 130.988, *p* < 0.001), and the interaction effect was significant (F = 95.411, *p* < 0.001). Similarly, for grip strength/weight index, there was no significant difference between the two groups (F = 2.836, *p* = 0.097), but significant improvements were observed over time in both groups (F = 20.839, *p* < 0.001), and the interaction effect was significant (F = 99.788, *p* < 0.001) (Table SI).

### Analysis of Barthel Index trends and RICU stay durations in VR-EM and control groups

**Fig. 3:**
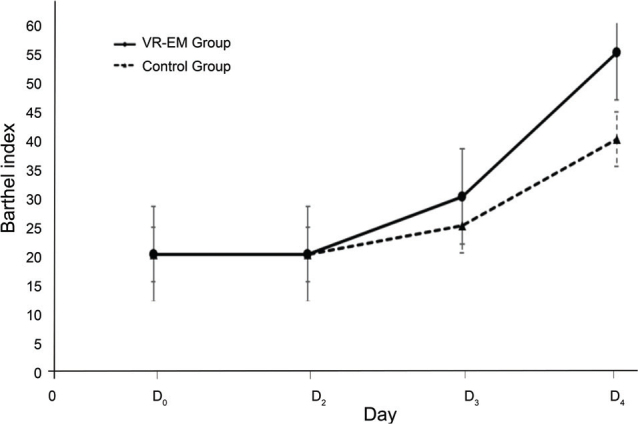
Barthel Index progression over time in VR-EM and control groups.

The change trend in the Barthel Index at baseline and within 6 days of intervention was also compared between the 2 groups. The median Barthel Index values for each time point of both groups were selected, and a line chart illustrating the relationship between the Barthel Index and time (D0~D6) was created using Excel, as shown in Fig. 3. Analysis of the Barthel Index trend in both groups indicated that the VR-EM group had a faster recovery rate regarding self-care ability than the control group. A linear mixed model was used to analyse the changing trend of Barthel Indexes in both groups within 1 week of intervention (Table IV).

**Table IV:** Comparison of the change trend of Barthel Index between the 2 groups within 6 days

Measurement index	Intergroup factor		Intra-group factors		Interaction	
	*F*	*p*-value	*F*	*p*-value	*F*	*p*-value
Barthel Index	1.547	0.216	37.849	< **0.001**	6.555	**0.013**

1. Intergroup factor analysis: there was no significant difference in Barthel Index between the VR-EM group and the control group (*p* > 0.05).

2. Intra-group factor analysis: the Barthel Index of patients in the VR-EM group and the control group had statistical significance at different time points (*p* < 0.001).

3. Interaction analysis: with the change of time, the effect of intervention still existed. There was significant difference in the Barthel Index between the VR-EM group and the control group (*p* < 0.001). The items shown in bold indicate statistically significant differences between the VR-EM group and the control group (*p* < 0.05). These results highlight the areas where the VR-based exercise intervention had a measurable and significant impact compared to the traditional exercise methods.

Additionally, the duration of RICU mechanical ventilation, RICU hospital stay, and the outcomes of both groups were compared. There was no significant difference in the duration and outcome of RICU mechanical ventilation between the 2 groups (VR-EM vs control: 5 vs 8 days) (*p >* 0.05). However, a significant difference was observed in the length of hospital stay in RICU between the 2 groups (VR-EM vs control: 11 vs 15 days) (*p* < 0.05). The VR group had a shorter ICU stay, a significant consequence of the intervention.

## DISCUSSION

This study is the first to compare the effectiveness of a virtual reality-based rehabilitation system with conventional exercise therapy on early mobilization in patients in the respiratory intensive care unit (RICU). Over the 1-week follow-up, the VR group demonstrated superior outcomes, including enhanced exercise duration, adherence, muscle strength, grip and handgrip strength, BMI, and self-care ability. These findings align with previous research suggesting that enhanced physical activity improves respiratory outcomes by reducing respiratory restrictions, increasing gas exchange efficiency, and shortening the duration of mechanical ventilation (19).

One key finding in this study was that the early active limb movement mode based on virtual reality enhances the early mobilization compliance of RICU patients. Virtual reality technology provides patients with an immersive experience and allows for interaction within a digital environment (20). This technology helps patients to divert their attention from negative feelings such as pain and discomfort during rehabilitation. Furthermore, it improves their engagement and enjoyment during rehabilitation treatment. For instance, previous studies by Jones et al. have demonstrated that watching virtual reality movies and playing games reduced pain levels in chronic patients (21). Similarly, the research of Hoffman et al. and Maani et al. indicated that virtual reality technology can alleviate pain in burn patients and serve as a non-pharmacological analgesic (22, 23). These results further reinforce that the virtual reality model fosters positive emotions among patients and encourages adherence to active exercise through entertaining and interactive rehabilitation experiences.

In VR-based early active limb movement, patients receive real-time feedback on visual motion, enabling posture adjustments. The immersive and engaging VR exercises, such as virtual piano playing for wrist stretches and virtual games for shoulder and elbow movements, highlight clinician–patient interactions and encourage patients to adhere to the exercise routine. These activities are facilitated by somatosensory devices that track movements and display mimicking virtual arms on-screen. For instance, in “Virtual Piano”, patients play melodies by pressing keys and completing wrist-stretching exercises with at least 20 stretches required per musical piece. Moreover, the VR system’s screen recording function gives medical staff a detailed understanding of patients’ rehabilitation progress and allows patients to observe their improvements, boosting their confidence and motivation. In contrast, the control group performed early active exercises using traditional methods taught independently by nurses or respiratory therapists in the Respiratory Intensive Care Unit (RICU). However, many patients found it difficult to maintain motivation without supervision, leading to lower adherence compared with the VR group. The interactive and immersive experiences provided by VR technology likely contributed to the observed quicker improvements in the VR group.

In this study, most patients in the RICU require respiratory support, such as endotracheal intubation or tracheotomy, which hampers effective nurse–patient communication. According to the cohort study by Freeman-Sanderson et al., patients using voice valves during weaning can tolerate more than 30 min of exercise, significantly improving their early mobilization ability (24). The study suggests that voice valves can alleviate patients’ feelings of isolation and enhance their cooperation with treatment. However, it is essential to note that the use of voice valves may lead to lung collapse and atelectasis. As a result, this method is suitable only for patients whose spontaneous breathing has significantly recovered (24). In this study, the virtual handwriting board function assists nurse–patient communication. It proves to be a safe and effective method with minimal patient burden. Patients need to use only their fingers to write on a flat desktop, and the computer can recognize their writing. This capability allows patients to express their needs, facilitating communication with nurses. Moreover, nurses can understand the true feelings of patients during the exercise process and take targeted measures accordingly.

Virtual reality technology has shown superior rehabilitation effects in various populations, including stroke and Parkinson’s patients, by improving compliance and physical function. Similarly, our study demonstrated the efficacy of VR-based limb movement in RICU patients, supporting its potential in critical care settings (25, 26). Kho and colleagues reported the feasibility of using interactive video games instead of traditional methods for early mobilization in ICU patients (14). In recent years, the application of electronic games and virtual reality systems in medical rehabilitation has increased (25). Some researchers have speculated that virtual reality systems may enhance the rehabilitation of motor function by activating and establishing neural connections between the prefrontal cortex and motor cortex (27).

Many studies have shown that the high economic burden of ICU may be an important reason for patients’ non-cooperation and refusal of treatment (28). VR-based early mobility therapy resulted in shorter hospital stays and improved grip strength (although not significantly different from bodyweight). At the same time, the Barthel Index of the VR group was more significant. All of these show that an early activity strategy based on VR for patients in ICU and daily life after discharge is likely helpful; it can reduce the economic burden on patients, prompting them to cooperate more with treatment.

In this study, real-time limb motion capture and feedback can provide clear goal orientation for patients, thereby helping them adhere to rehabilitation training. Hodgson et al. and Schaller et al. implemented goal-oriented early mobilization programmes for critically ill patients, and those who participated in these programmes improved their exercise ability (29, 30). Similarly, in a previous study, early goal-oriented activities were safe and effective for rehabilitating RICU patients (31). The patients who received early goal-oriented activities demonstrated higher muscle strength, grip strength, and self-care ability than the control group. These findings validate that patients who accept a target-oriented approach can expedite their rehabilitation process and reduce the duration of ICU hospitalization after early mobilization, which aligns with the conclusions of various domestic and international studies that have examined the effect of early mobilization in the ICU setting.

While the numerical values and composition ratios related to mechanical ventilation favour the virtual reality group compared with the control group, no statistical difference was observed. Some researchers have reported that early mobilization in the ICU can shorten the duration of mechanical ventilation (1), but recent meta-analyses have not confirmed the effectiveness of early mobilization in this outcome (32). The researchers speculate that the lack of consistency may be attributed to the study’s variation in patient populations and the different circumstances surrounding mechanical ventilation. Similarly, a meta-analysis on the effect of early mobilization in the ICU indicated that early mobilization did not reduce in-hospital mortality (33), which concurs with the results of this study.

This study highlights the potential of VR-based early active limb movement to enhance rehabilitation outcomes in RICU patients. However, limitations such as the small sample size, single-centre design, and short follow-up period raise concerns regarding generalizability. Multicentre studies with larger samples and extended follow-up are needed to validate these findings.

The gaming system’s limited functionalities in human–computer interaction and motion capture should be improved, along with developing more engaging rehabilitation games. Additionally, future research should include respiratory function testing to confirm these results and explore long-term impacts through extended observational studies.

In conclusion, compared with traditional exercise methods, the early active limb movement mode utilizing virtual reality for 1 week shows the potential to enhance exercise perception and self-confidence among RICU patients. Additionally, it significantly increases the average daily exercise time and out-of-bed exercise duration, leading to improved exercise compliance and accelerated recovery in muscle strength (MRC score, *p* = 0.006). The findings also suggest that this mode may reduce the duration of RICU hospitalization. Overall, the study highlights the potential of virtual-reality-based early active limb movement mode as a feasible and beneficial approach in rehabilitating RICU patients. Further multicentre studies with larger sample sizes and longer follow-up periods are needed to validate these findings and explore the long-term impact of VR-assisted exercise on patient outcomes.

## Supplementary Material

EFFECTIVENESS OF VIRTUAL REALITY ASSISTED ACTIVE LIMB MOVEMENT EXERCISES FOR PATIENTS IN THE RESPIRATORY INTENSIVE CARE UNIT: A RANDOMIZED PILOT STUDY
