# Supplementary material for: EFFECTIVENESS OF VIRTUAL REALITY ASSISTED ACTIVE LIMB MOVEMENT EXERCISES FOR PATIENTS IN THE RESPIRATORY INTENSIVE CARE UNIT: A RANDOMIZED PILOT STUDY
Source: J Rehabil Med. 2025 Jun 3;57:28399. doi: 10.2340/jrm.v57.28399 (PMC12160588; doi:10.2340/jrm.v57.28399)
Supplement: EFFECTIVENESS OF VIRTUAL REALITY ASSISTED ACTIVE LIMB MOVEMENT EXERCISES FOR PATIENTS IN THE RESPIRATORY INTENSIVE CARE UNIT: A RANDOMIZED PILOT STUDY [file JRM-57-28399-s1.pdf]

Supplementary material has been published as submitted. It has not been copyedited, or typeset by Journal of Rehabilitation Medicine

Table SI. Comparison of the changing trend of muscle strength, grip strength and body mass index between the two groups within one week of intervention

Measurement index	Intergroup factor		Intra-group factors		Interaction	
	F	*P*	*F*	*P*	*F*	*P*
**MRC-Score**	7.921	**0.006**	26.159	**< 0.001**	50.10	**< 0.001**
**Grip strength**	1.430	0.235	130.988	**< 0.001**	95.411	**< 0.001**
**Grip strength/ weight index**	2.836	0.097	20.839	**< 0.001**	99.788	**< 0.001**
